# The relation of cluster headache to alexithymia, depression, and anxiety

**DOI:** 10.1055/s-0044-1791516

**Published:** 2024-11-03

**Authors:** Dilek İşcan, Cansu Dal

**Affiliations:** 1 Nigde Omer Halisdemir University, Faculty of Medicine, Department of Neurology, Nigde, Turkey.; 2 Mugla Sitki Kocman University, Institute of Health Sciences, Department of Physiotherapy and Rehabilitation, Mugla, Turkey.

**Keywords:** Cluster Headache, Affective Symptoms, Depression, Anxiety, Limbic System, Cefalalgia Histamínica, Síntomas Afectivos, Depresión, Ansiedad, Sistema Límbico

## Abstract

**Background** The pathophysiology of cluster headaches (CHs) involves the trigeminovascular system, the parasympathetic nervous system, and the hypothalamus. Because of the affected hypothalamus, there may be limbic system involvement in CH.

**Objective** To investigate the relationship between depression, anxiety, and alexithymia in CH, to demonstrate that the limbic system is affected.

**Methods** A total of 18 patients with CHs who were outside of cluster period and 18 healthy controls were included. Participants were administered the Beck depression inventory (BDI), Beck anxiety inventory (BAI), and Toronto alexithymia scale-20 (TAS-20).

**Results** Patients with CHs had significantly higher rates of alexithymia ( *p* = 0.003) and depression ( *p* = 0.014) than controls. There was no significant difference in anxiety levels ( *p* = 0.297) between groups.

**Conclusion** It was shown that, in addition to previously identified psychiatric disorders, alexithymia can accompany CHs in patients.

## Resumen

**Antecedentes** La fisiopatología de la cefalea en racimos (CER) incluye el sistema trigeminovascular, el sistema nervioso parasimpático y el hipotálamo. Debido a la afectación del hipotálamo, puede haber una afectación del sistema límbico en la CER.

**Objetivo** Este estudio pretendía investigar la relación entre depresión, ansiedad y alexitimia en CER para demostrar que el sistema límbico está afectado.

**Métodos** Se incluyeron en el estudio 18 pacientes con CER que se encontraban fuera del periodo de cluster y 18 sujetos control sanos. Se administró a los participantes el inventario de depresión de Beck (IDB), el inventario de ansiedad de Beck (IAB) y la escala de alexitimia de Toronto-20 (TAS-20).

**Resultados** Los pacientes con CER presentaban tasas significativamente más altas de alexitimia ( *p* = 0.003) y depresión ( *p* = 0.014) que los controles. No había diferencias significativas en los niveles de ansiedad ( *p* = 0.297) entre los grupos.

**Conclusión** En pacientes diagnosticados de CER, se demostró que además de los trastornos psiquiátricos previamente identificados, la alexitimia también puede acompañar a la enfermedad.

## INTRODUCTION

Cluster headaches (CH) are an autonomic cephalalgia characterized by severe, unilateral attacks lasting 15 to 180 minutes that can occur up to eight times a day. Painful periods persist for weeks, followed by painless periods that span months or years. Attacks are accompanied by cranial autonomic symptoms such as lacrimation, nasal congestion, rhinorrhea, eyelid oedema, and ptosis, as well as agitation or a feeling of restlessness. 1

The average onset age is 30 years, typically beginning between the ages of 20 and 40, and males are three to four times more affected than females. 2 3 The lifetime incidence rate is 124 per 100,000. 4 Diagnosis is done according to the criteria of the International Classification of Headache Disorders 3 ^rd^ edition (ICHD-3), developed by the Headache Classification Committee of the International Headache Society. 5 Differential diagnoses include short-lasting, unilateral, neuralgiform headache attacks with conjunctival injection and Tearing (SUNCT), hemicrania continua, migraine, and trigeminal neuralgia. 5

Acute treatment may include high-flow oxygen therapy, triptans, corticosteroids, lidocaine, whereas prophylactic treatment options include drugs like verapamil, lithium, topiramate, and galcanezumab. 3 It is believed that CH results from the involvement of the trigeminovascular system, parasympathetic nervous system, and hypothalamus. 6 The hypothalamus is part of the limbic system, which also includes the insula, amygdala, hippocampus, cingulate gyrus, prefrontal cortex, and thalamus. The limbic system also has an important function role in social cognition. 7

Alexithymia is defined as a personality feature characterized by a deficiency in cognitive processes for describing, differentiating, and conveying emotional states, also known as emotional agnosia. 8 According to research, alexithymia is associated with several mental and physical health problems, including somatoform disorders, depression, and medically unexplained symptoms. 9 10 Depression and anxiety are more frequent in patients with CH than in those without. 11 The hypothalamus is essential to psychiatric comorbidities in this condition. 12

Often being called “suicide headache” due to its severity and resistance to treatment, CH has not yet received adequate research into the relationship between alexithymia, depression, and anxiety. The present study aimed to investigate this relationship in CH, which had been previously underexplored in the literature.

## METHODS

The study was conducted at the Neurologic Outpatient Clinic of Niğde Ömer Halisdemir University Training and Research Hospital. The study was approved by the local Ethics Committee (no. 2024/33). Before the study, all participants were thoroughly briefed, then gave their written informed consent.

### Study design

This cross-sectional observational included 18 patients diagnosed with CH using the ICHD-3 diagnostic criteria, 5 who were not in the middle of a headache episode. The control group consisted of 18 healthy adults of similar age and gender, free of any psychiatric disorders, migraine, CH, or other primary headaches, and not taking any medications, selected from among the hospital visitors, primarily family members.

After both groups were identified, the scores for depression, anxiety, and alexithymia for each group were calculated using the Beck Depression Inventory (BDI), Beck Anxiety Inventory (BAI) and Toronto Alexithymia Scale-20 (TAS-20), respectively.

### Beck depression inventory (BDI)

The BDI is a scale that assesses the severity of depression. The Turkish version was validated by Şahin et al. 13 The assessment includes 21 domains, each with a score ranging from 0 to 3. A score of 0 to 9 is considered normal, whereas a score of 10 to 16 indicates mild, 17 to 29 moderate, and 30 to 63 severe depression. 14

The reliability of the BDI was examined in this study using item analysis and split-half techniques, which yielded correlation coefficients of r = 0.80 and 0.74, respectively. The concurrent validity of the scale was examined using the Minnesota Multiphasic Personality Inventory-Depression (MMPI-D) scale as the criterion. The Pearson correlation coefficient for the two scales was r = 0.50. These reliability and validity coefficients are comparable with previous studies conducted for this purpose. 15

### Beck anxiety inventory (BAI)

Ulusoy et al. conducted a Turkish validity and reliability research on the BAI, which is used to evaluate the degree of anxiety. 16

The assessment includes 21 topics, each with a score ranging from 0 to 3 points. Mild anxiety symptoms are scored between 8 and 15 points, whereas moderate anxiety symptoms range between 16 and 25 points. A score between 26 and 63 points indicates severe anxiety symptoms. 17

The BAI showed strong internal consistency (α = 0.93). The item-total correlations ranged between 0.45 and 0.72. The exploratory factor analysis identified two factors. Overall, the BAI is considered a reliable and accurate measure of anxiety in Turkish psychiatric populations. 17

### Toronto alexithymic scale-20 (TAS-20)

Güleç et al. evaluated alexithymia in terms of the validity and reliability of the Turkish translation of the 20-point Toronto alexithymia scale (TAS-20) from 2017. 18 The scale spans from 1 to 5 and includes four subdimensions: ability to distinguish and recognize emotions, thinking about external events, capability to express emotions, and imagination. Those with TAS ≤ 51 showed no symptoms of alexitymia, those with 52 to 60 were considered as likely alexithymic, and those with ≥ 61 were considered absolutely alexithymic. 19

The Turkish TAS-20 showed a three-factor model. The Cronbach α for the entire scale was 0.78, whereas the three subscales (factors 1–3) were 0.80, 0.57, and 0.63, respectively. Three of the four goodness-of-fit criteria met the standards for the adequacy-of-fit criterion. The parameter estimates for the items and the correlation between the three factors were as follows: 0.53 between factors 1 and 2, 0.12 between 1 and 3, and 0.36 between 2 and 3. All items (except 18 and 20) have significant correlations with the total score, with values ranging from 0.22 to 0.48. The Turkish TAS-20 factor analysis yielded a three-factor structure that was consistent with the original scale, and the translation had good internal consistency. Thus, this scale is a valid construct for Turkish research. 19

### Statistical analysis

Statistical analysis of the data obtained from the study was performed using the SPSS Statistics for Windows, version 22.0 (SPSS, IBM Corp., Armonk, NY, USA) . The Kolmogorov-Smirnov test was used to determine whether the variables were normally distributed. Mean and standard deviation (mean ± SD) were used for normally distributed variables, whereas median and interquartile range (IQR) values were provided for nonnormally distributed variables. The Chi-squared test was used to assess qualitative independent variables, such as gender and education level. To compare data obtained from the CH and control groups, the independent *t* -test was used for variables with normal distributions, and the Mann-Whitney U test for variables with nonnormal distributions. The data were analyzed using the Chi-squared test and Bonferroni-adjusted *p* -values, with the statistical significance set at *p* < 0.05.

## RESULTS

The CH group of 18 patients had a mean age of 34.78 ± 8.981, with 11.1% (n = 2) being female. The control group, which included two female participants, had a mean age of 35.28 ± 9.138 ( *p* = 0.869). As shown in Table 1 , there was no statistically significant difference in education levels across the groups. When asked about the frequency of CH attacks, most participants (66.66%) reported experiencing them once a year. The remaining six (33.33%) reported experiencing attacks twice a year.

**Table 1:** Demographic data of groups and attack frequency of the patients

		Cluster headache	Control group	*p* -value
Age, years (mean ± SD)		34.78 ± 8.98	35.28 ± 9.13	0.896
Gender, n (%)	Female	2 (11%)	2 (11%)	1.00
	Male	16 (89%)	16 (89%)	
Education level, n (%)	Secondary or high school	9 (50%)	7 (39%)	0.516
	Undergraduate, graduate, or bachelor's degree	9 (50%)	11 (61%)	
Attack frequency, n (%)	Once a year	12 (66.7%)		
	Twice a year	6 (33.3%)		

Abbreviations: n, number; SD, standard deviation.

The CH group had a significantly higher depression score (BDI, 10.78 ± 6.88) than the control group (4.73 ± 1.11, *p* < 0.05), and significantly higher TAS-20 scores (55.61 ± 5.66) than the control group (45.50 ± 3.36; *p* < 0.05). Although the control group had higher anxiety scores (BAI) than the CH group, there was no statistically significant difference in BAI scores between the two groups ( *p* > 0.05).

As shown in Table 2 , according to the TAS-20, there were 10 (55.55%) participants with probable alexithymia and two (11.11%) with confirmed alexithymia among CH patients. Only two (11.11%) participants of the control group exhibited probable alexithymia. According to the BDI, seven participants (38.88%) had mild depression, and four (22.22%) had moderate depression in the CH group, while in the control group three (16.66%) participants had mild depression. According to the BAI, in the CH group, 15 (83.33%) participant had minimal anxiety, two (11.11%) had mild anxiety, and one (5.55%) had moderate anxiety. In the control group, 13 (72.22%) participants had minimal anxiety, and five (27.77%) had mild anxiety.

**Table 2:** Comparison of depression, anxiety, and alexithymia levels between groups

Parameters		Cluster headache n (%)	Control group n (%)	Total n (%)	*p* -value
Depression levels	Normal	7 (38.9)	15 (83.3)	22 (61.1)	0.014*
	Mild	7 (38.9)	3 (16.7)	10 (27.8)	
	Moderate	4 (22.2)	0 (0)	4 (11.1)	
Anxiety levels	Minimal	15 (83.3)	13 (72.2)	28 (77.8)	0.297
	Mild	2 (11.1)	5 (27.8)	7 (19.4)	
	Moderate	1 (5.6)	0 (0)	1 (2.8)	
Alexithymia levels	None	6 (55.6)	16 (88.9)	22 (61.1)	0.003*
	Possible	10 (33.3)	2 (11.1)	12 (33.3)	
	Definite	2 (11.1)	0 (0)	2 (5.6)	

Abbreviation: n, number. Notes: The Chi-squared test was used to determine statistical significance; * *p* < 0.05.

## DISCUSSION

In recent years, a growing amount of research has focused on the autonomic, behavioral and cognitive deficits that accompany the basic symptoms of neurological diseases. While executive function studies have been conducted in CH, research on social cognition remains limited. Furthermore, although the relationship between alexithymia and primary headaches, such as migraine and tension-type headaches, has been investigated, there was only one study conducted on the relationship between CH and alexithymia. 20 In the present study, we compared the levels of anxiety, depression, and alexithymia between patients with CH who had not experienced a cluster attack and healthy controls.

The CH group had significantly higher scores on depression and alexithymia. However, there was no statistically significant difference in anxiety between the groups. There were two (11.11%) participants with CH who showed definite alexithymia and 10 (55.55%) with probable alexithymia.

Although it is unclear whether nonspecific pathophysiological mechanisms of CH or headaches cause neuropsychological involvement, which has already been demonstrated in CH. In a study by Munoz et al., it was shown that individuals with CH exhibited an increase in cluster A personality traits compared to migraine patients. 21 Louter et al. found that those with CH are three times more likely to suffer lifelong depression. 22 It has been shown that over 90% of patients may experience not only restlessness during attacks but also various complicated and occasionally violent behaviors, including self-harming. 23 Rausa et al. found that feelings of anger were more intense in those with CH during attack periods. 24

Schenck et al. found that individuals with CH were angrier during attack periods than those without attacks or patients who had migraine attacks. 25 Compared to migraine patients with and without aura, as well as those with tension-type headache, individuals with CH struggle more with emotion and adaptation. 26 Participants with CH exhibited a more consistent lack of emotion, social interaction, identification and responsibility as compared with migraine with and without aura and tension-type headache. 26

Galli et al. found that the occurrence of alexithymia in patients with chronic and episodic migraine was unrelated to disease severity. 27 Neyal et al. showed higher levels of alexithymia in patients with tension-type headache. 28 Nevertheless, we were unable to find a literature review that addressed alexithymia in a large sample size of patients diagnosed with CH.

The limitations of this study include its small sample size and single-center recruitment tactic. The findings of this study must be supported by further research. Increasing sample size and recruiting patients from several clinics may provide more robust findings.

In conclusion, our research showed that CH is more than just a painful condition. It is a disorder characterized by alexithymia and previously reported psychiatric symptoms, supported by extensive statistical calculations. We recommend that the treatment approach for these patients be organized around examining psychological symptoms in addition to pain management.
